# Neural Stochastic Differential Equations with Neural Processes Family Members for Uncertainty Estimation in Deep Learning

**DOI:** 10.3390/s21113708

**Published:** 2021-05-26

**Authors:** Yongguang Wang, Shuzhen Yao

**Affiliations:** School of Computer Science and Engineering, Beihang University, Beijing 100191, China; szyao@buaa.edu.cn

**Keywords:** deep neural networks, neural stochastic differential equation, neural processes, uncertainty estimates

## Abstract

Existing neural stochastic differential equation models, such as SDE-Net, can quantify the uncertainties of deep neural networks (DNNs) from a dynamical system perspective. SDE-Net is either dominated by its drift net with in-distribution (ID) data to achieve good predictive accuracy, or dominated by its diffusion net with out-of-distribution (OOD) data to generate high diffusion for characterizing model uncertainty. However, it does not consider the general situation in a wider field, such as ID data with noise or high missing rates in practice. In order to effectively deal with noisy ID data for credible uncertainty estimation, we propose a vNPs-SDE model, which firstly applies variants of neural processes (NPs) to deal with the noisy ID data, following which the completed ID data can be processed more effectively by SDE-Net. Experimental results show that the proposed vNPs-SDE model can be implemented with convolutional conditional neural processes (ConvCNPs), which have the property of translation equivariance, and can effectively handle the ID data with missing rates for one-dimensional (1D) regression and two-dimensional (2D) image classification tasks. Alternatively, vNPs-SDE can be implemented with conditional neural processes (CNPs) or attentive neural processes (ANPs), which have the property of permutation invariance, and exceeds vanilla SDE-Net in multidimensional regression tasks.

## 1. Introduction

Deep learning models have achieved great success in many fields, such as image classification [1], computer vision [2], machine translation [3], and reinforcement learning [4]. However, in key fields where safety is at stake, such as in medical diagnoses or autonomous vehicles, the uncertainty estimation of deep learning models is essential for decision making in order to avoid dangerous accidents. Existing studies have shown that deep neural networks (DNNs) models are usually miscalibrated and overconfident in their predictions, which can result in misleading decisions for out-of-distribution (OOD) samples, so it is very important to add credible uncertainty estimates to the predicted values [5].

Bayesian neural networks (BNNs) methods were once regarded as a gold standard for uncertainty estimation in machine learning models [6,7], and the recent benchmark Bayesian method applies a backpropagation-compatible algorithm for learning a probability distribution on the weight of a neural network, which is called Bayes by Backpropagation (BBP) [8]. However, Bayesian methods are very inefficient when performing posterior inference in DNNs with a large number of parameters. On the one hand, in order to improve efficiency, the existing studies adopt the linear subspace feature extracting method of principal component analysis (PCA) in order to construct the parameter subspace of DNNs for a Bayesian inference [9], and the curve parameter subspace method is proposed to build a rich subspace containing diverse, high-performing models [10]. Meanwhile, the latest incremental kernel PCA (InKPCA) approach applies kernel PCA to extract higher order statistical information from DNNs’ parameter space, and achieves more accurate results than the PCA and curve subspace inference methods [11]. On the other hand, to approximate the Bayesian inference method, dropout in NNs can be interpreted as an approximation of the Gaussian process (GP), and dropout variational inference (DVI) can be an approximate Bayesian inference approach for large and complex DNN models [12].

Non-Bayesian methods are also studied for uncertainty estimation in DNNs models. For example, the ensemble modeling approach trains several DNNs models with diverse initialization seeds, and uses the predicted values for uncertainty estimation [13]. Meanwhile, if DNNs are trained with a stochastic gradient descent (SGD), the training procedure can average multiple points along the trajectory of the SGD in order to construct a stochastic weight averaging (SWA), which produces much broader optima than an SGD [14]. Due to the dynamics of training DNNs with SGD-like optimizers having some properties similar to overfitting, in which the predicted values are overconfident, the pointwise early stopping algorithm for confidence scores selectively estimates the uncertainty of highly confident points in deep neural classifiers [15]. Additionally, the Monte Carlo dropout (MC-dropout) method casts dropout training in DNNs as approximate Bayesian inference and samples at the test phase, and then applies variance statistics for multiple dropout-enabled forward passes [16].

Most of the uncertainty estimation models mentioned above mainly consider the predictive uncertainty that comes from models and their training processes, known as “epistemic uncertainty” [16]. However, further predictive uncertainty derives from natural randomness such as noisy data and labels, class overlap, incomplete features, and other unknown factors; this is known as “aleatoric uncertainty” [17], which is inherent in the task and cannot be explained away with further data. Fortunately, a unified Bayesian deep learning framework has been proposed by [17], which can help us to capture an accurate understanding of aleatoric and epistemic uncertainty for per-pixel depth regression and semantic segmentation tasks. Forward passes in DNNs can be considered to be state transformations of a dynamical system, which can be defined by a neural-network-parameterized ordinary differential equation (ODE) [18]. An ODE is a deterministic expression, so it cannot obtain the epistemic uncertainty message.

**Figure 1:**
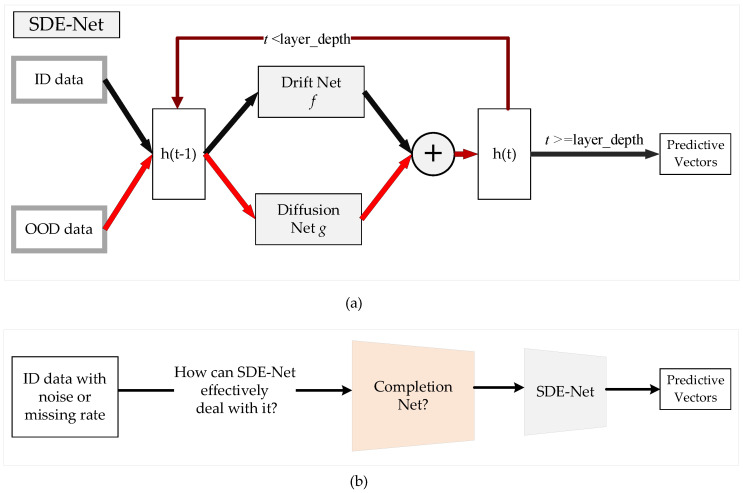
Illustration of SDE-Net and the problems it faces. (**a**) Components of SDE-Net. For ID data, SDE-Net is dominated by the drift net in order to achieve good predictive accuracy. For OOD data, SDE-Net is dominated by the diffusion net in order to generate high diffusion for characterizing model uncertainty. (**b**) Flowchart explaining how to resolve the problems faced by SDE-Net.

Recently, a novel SDE-Net for uncertainty estimation of DNNs has been proposed to capture epistemic uncertainty using Brownian motion or the Wiener process [19,20], which are widely used to model uncertainty or randomness in mathematics, physics, economics, and other disciplines [21,22]. SDE-Net uses two separate neural networks (NNs): the drift net *f* is designed to control the system in order to achieve a good predictive accuracy for in-distribution (ID) data, while the diffusion net *g* is used to control the variance of the Brownian motion based on the ID or OOD regions. SDE-Net can not only explicitly model aleatoric uncertainty and epistemic uncertainty in its predictions for classification and regression tasks, but also does not need to specifically model prior distributions and infer posterior distributions as in BNNs. SDE-Net can achieve good performance and uncertainty estimation between ID and OOD data; however, in practice, ID data are generally encountered with noise or high missing rates. The purpose of this paper is to explore how to effectively deal with ID data with noise or high missing rates for SDE-Net. Our idea is to first fix the noisy ID data with a completion net, and then process the completed data with SDE-Net for uncertainty estimation. The components of SDE-Net are described in Figure 1a, and the resolution of SDE-Net’s problems is explained in Figure 1b. Figure 1 shows that SDE-Net lacks consideration of the general situation in a wider field—that is, ID data with noise or high missing rates in practice.

To handle OOD data, existing studies either use confidence scores to determine whether samples are ID or OOD [23], or use a new confidence loss on a sharp predictive distribution for ID data and a flat predictive distribution for OOD data [24]. These new methods adopt loss functions to produce deterministic results. However, Dirichlet distribution—which allows high uncertainty for OOD data, but is only applicable to classification tasks—is parameterized over categorical distributions [25].

To handle noisy ID data, recent contributions indicate that the regularization technique dropout can degrade DNNs’ training performance on noisy data without compromising generalization on real data [26]. More importantly, the dropout method has been proven to be a Bayesian approximation, and can represent model uncertainty in deep learning [27]. Moreover, the latest study establishes the first benchmark of controlled real-world label noise from the internet, and can conduct the largest study into understanding DNNs trained on noisy labels across different settings—such as noise levels, noise types, network architectures, and training settings. Thus, this method can be studied further for uncertainty estimation in deep learning. Of course, there are many other methods that deserve further study for uncertainty estimation, such as label cleaning/correction, example weighting, data augmentation, etc.

However, NP methods can combine the advantages of GPs in flexibility and neural networks with high precision; thus, we propose to add neural processes (NPs) [28] to SDE-Net in order to improve the its accuracy with noisy ID data, where NP variants include conditional neural processes (CNPs) [29], attentive neural processes (ANPs) [30], and convolutional conditional neural processes (ConvCNPs) [31]. We use the abbreviation vNPs to represent the NP family, which includes vanilla NPs and NP variants. The combination of vNPs and SDE-Net (vNPs–SDE) is motivated by the permutation invariance or equivariance properties of vNPs for ID data with noise or high missing rates in SDE-Net.

CNPs define distributions over functions given a set of observations, and the dependence of a CNP on the observations is parameterized by a neural network, which is invariant under permutations of its inputs. Meanwhile, vanilla NPs are a generalization to other NP variants, and the vanilla NPs generate fixed-length latent variables via a permutation invariant function. Moreover, ANPs apply an attention mechanism in order to compute the weights of each key with respect to the query [32]. ConvCNPs can model translation equivariance in the data and embed datasets into an infinite-dimensional function space.

Although convolutional neural networks (CNNs) can also apply translation equivariance to time series or image tasks [33,34], the translation equivariance of CNNs models is not straightforward to generalize to the NP family in the same way. This is because CNNs models need image pixels in order to form a regularly spaced grid, while NPs perform on partially observed context sets in order to embed them into a finite-dimensional vector space.

To summarize, there are two main contributions of this paper:

- Considering the translation equivariance properties of ConvCNPs, the implementation of the vNPs–SDE model with ConvCNPs can effectively handle ID data with missing rates for 1D regression and 2D image classification tasks.
- Applying the property of permutation invariance, the implemented vNPs–SDE model with CNPs or ANPs surpasses BBP, MC-dropout, and vanilla SDE-Net in multidimensional regression tasks with high missing rates by most metrics.

The rest of this paper is organized as follows: Section 2 describes materials and methods for uncertainty estimation in deep learning models with noisy ID data. Section 3 presents the implementation of the proposed vNPs–SDE model for different tasks in deep learning. Section 4 demonstrates the results of ConvCNPs–SDE model for 1D regression and 2D image classification tasks with high missing rates, and of the CNPs–SDE and ANPs–SDE models for multidimensional regression tasks with high missing rates. Section 5 presents the discussion of the experimental results, and Section 6 presents the conclusions and implications for future work.

## 2. Materials

In this section, we mainly introduce the concepts to be used in this paper, such as stochastic processes and NPs and the relationship between them.

### 2.1. Definition of the Neural Processes Family

Neural processes as stochastic processes. For each finite sequence $x_{1:n}$ = ($x_{1},\cdots ,x_{n}$) with $x_{i}\in X$, the finite-dimensional marginal joint distribution over the function *f* values can be defined as $Y_{1:n}≔\left(f\left(x_{1}\right),\cdots ,\;f\left(x_{n}\right)\right)$. For example, in the popular Gaussian processes (GPs) model, the joint distributions are multivariate Gaussian distributions parameterized by a mean and a covariance function.

As stated by the Kolmogorov extension theorem [20], two necessary conditions—(finite) exchangeability, and consistency for marginal joint distributions $\rho_{x_{1:n}}$ of $\left(f\left(x_{1}\right),\cdots ,\;f\left(x_{n}\right)\right)$—can be sufficient to define a stochastic process.

**Property** **1** (Exchangeability) [28]. *If for each finite n element,* $x_{1:n}$*, π represents a permutation of* (1,⋯, n)*, then:*

(1) $$\rho_{x_{1:n}}{(y}_{1:n})≔\rho_{x_{1},\cdots x_{n}}{(y}_{1},\cdots ,y_{n}{)=\rho }_{x_{\pi (1)},\cdots ,\;x_{\pi (n)}}{(y}_{\pi (1)},\cdots ,y_{\pi (n)}{)=:\rho }_{{\pi (x}_{1:n})}{(\pi (y}_{1:n}))$$

**Property** **2** (Consistency) [28]. *If* 1≤m≤n*, and we marginalize out a part* $\left(y_{m+1},\cdots ,\;y_{n}\right)$ *of the sequence* $\left(y_{1},\cdots ,\;y_{n}\right)$ *of* Y*, the resulting marginal distribution is the same as that defined in the original sequence. That is:*

(2) $$\rho_{x_{1:m}}{(y}_{1:m})=\int \rho_{x_{1:n}}{(y}_{1:n}{)dy}_{m+1:n}$$

Exchangeability and consistency can define a stochastic process, assuming a stochastic process *f* can be parameterized by a global and high-dimensional random vector *z*, so we can define a generative model:

(3) $$p({z,\;y}_{1:n}|\;x_{1:n})=p\left(z\right)\prod_{i=1}^{n=1}N\left(y_{i}|g(x_{i}{,\;z),\sigma }^{2}\right)$$

NPs contain a latent variable *z* to capture stochastic process *f* and global uncertainty. An NP model is composed of three key components:

(1) Encoder: The encoder *E* of NPs has two paths—a deterministic path and a latent path. In the deterministic path, each context pair ${\left(x,y\right)}_{i}$ is passed through a multi-layer perceptron $\left({\mathrm{MLP}}_{\theta }\right)$ to produce a deterministic representation $r_{i}$. In the latent path, a latent representation $s_{i}$ is generated by passing through each context pair ${\left(x,y\right)}_{i}$ to another ${\mathrm{MLP}}_{\psi }$. Thus, the purpose of encoder *E* is to convert the input space into deterministic or latent representation space, where the input space represents *n* context points $C={\left\{{\left(x,y\right)}_{i}\right\}}_{i=1}^{n}$, and the representation space produces $r_{i}={\mathrm{MLP}}_{\theta }\left({\left(x,y\right)}_{i}\right)$ and $s_{i}={\mathrm{MLP}}_{\psi }\left({\left(x,y\right)}_{i}\right)$ for each of the pairs ${\left(x,y\right)}_{i}$.
(2) Aggregator: Aggregator *a* aims to summarise the *n* global representations $r_{1\ldots n}$ and $s_{1\ldots n}$. The simplest operation of aggregator *a* is the mean function $m=a\left(m_{i}\right)=\frac{1}{n}\overset{n}{\underset{i=1}{\sum }}m_{i}$, which can ensure order invariance and perform well in practice. For the deterministic path, *a* is applied to $r_{1\ldots n}$ to produce the deterministic code $r_{C}$. For the latent path, however, we are interested in achieving an order-invariant global latent representation, so we apply *a* to $s_{1\ldots n}$ to produce the latent code $s_{C}$, which can parameterize the normal distribution $z\;\sim \;N\left(\mu \left(s_{C}\right),\;I\sigma \left(s_{C}\right)\right)$ for the latent path.
(3) Decoder: In decoder *D*, the sampled global latent variables z and $r_{C}$ are concatenated alongside the new target locations $x_{T}$ as inputs, and finally passed through *D* to produce the predictions ${\hat{y}}_{T}=D(x_{T},\;r_{C},\;z)$ for the corresponding values of $f\left(x_{T}\right){=y}_{T}$. We parameterize decoder *D* as a neural network.

NPs are a generalization of ANPs and CNPs, but CNPs lack a latent variable z that allows for global sampling.

**Figure 2:**
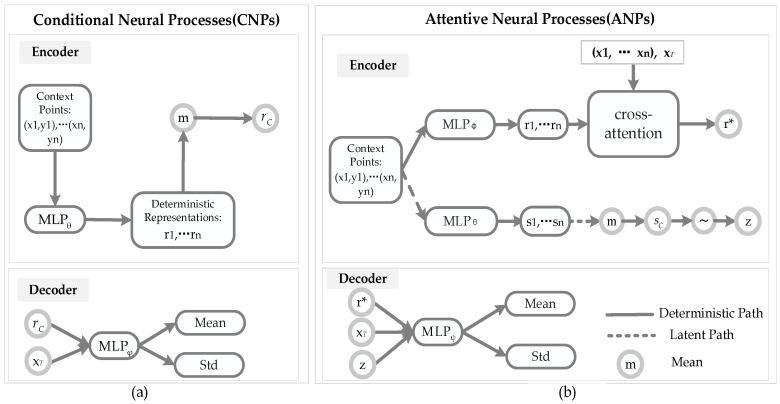
Architectures of CNPs and ANPs. (**a**) Components of a CNP model: the encoder of CNPs is composed of a deterministic path to generate a representation *r*; the decoder of CNPs uses the *r* and target $x_{T}$ to produce the mean and the Std. (**b**) Components of an ANP model: the encoder of ANPs is composed of a deterministic path to generate a representation $r^{*}$ and a latent path to generate latent variable *z*; the decoder of ANPs uses the $r^{*}$, *z*, and target $x_{T}$ to produce the mean and the Std.

The architectures of CNPs and ANPs are described in Figure 2.

Considering the CNPs model in Figure 2a, compared with the NPs and ANPs models, CNPs lack a latent path, and only produce the deterministic representation $r_{i}={\mathrm{MLP}}_{\theta }\left({\left(x,y\right)}_{i}\right)$ for each of the context pairs ${\left(x,y\right)}_{i}$ in the encoder. Then, the aggregator *a* of the CNPs summarises the *n* global representations $r_{1\ldots n}$ to produce the deterministic code $r_{C}$. Finally, $r_{C}$ is concatenated alongside the new target locations $x_{T}$ as an input, and passed through the neural network ${\mathrm{MLP}}_{\varphi }$ to produce the predictions ${\hat{y}}_{T}={\mathrm{MLP}}_{\varphi }(x_{T},\;r_{C})$ in the decoder.

Considering the ANPs model in Figure 2b, compared with the NPs model, ANPs add an attention mechanism to increase the accuracy of the NPs. Assume that there are *n* key–value pairs $\left(K,\;V\right)={\left(x_{i}{,\;y}_{i}\right)}_{i=1}^{n}$, where $K\in {\mathbb{R}}^{n\times d_{k}},\;V\in {\mathbb{R}}^{n\times d_{v}}$, and *m* query $x_{T}=Q\in {\mathbb{R}}^{m\times d_{k}}$. There are several attention mechanisms, such as uniform, laplace, dot product, and multihead:

- $\mathrm{Uniform}\left(Q,K,V\right)≔\frac{1}{n}\overset{n}{\underset{i=1}{\sum }}x_{i}$;
- $\mathrm{Laplace}\left(Q,K,V\right)≔\mathrm{WV}\in {\mathbb{R}}^{n\times d_{v}}$, $W_{i\cdot }≔\mathrm{softmax}\left({\left(-∥Q_{i}-K_{j}∥{}_{1}\right)}_{j=1}^{n}\right)\in {\mathbb{R}}^{n}$;
- DotProduct(Q,K,V)≔softmax(QKTdk)V∈ℝm×dv;
- $\mathrm{MultiHead}\left(Q,K,V\right)≔\mathrm{concat}\;\left({\mathrm{head}}_{1},\cdots ,{\mathrm{head}}_{H}\right)W\in {\mathbb{R}}^{m\times d_{v}}$,

where ${\mathrm{head}}_{h}≔\mathrm{DotProduct}\left({\mathrm{QW}}_{h}^{\;Q},{\mathrm{KW}}_{h}^{\;K},{\mathrm{VW}}_{h}^{V}\right)\in {\mathbb{R}}^{m\times d_{v}}$.

Compared with NPs, the advantage of an ANPs model is to incorporate attention mechanisms into the NPs model. In short, self-attention of the encoder in the deterministic and latent paths can be implemented via a multilayer perceptron (MLP), which is applied to the context points to get the representations $r_{1\ldots n}$, and then target input $x_{T}$ attends to $r_{1\ldots n}$ and $x_{1\ldots n}$ with cross-attention to predict the target output r*. In the aggregator and the decoder, ANPs and NPs have similar operations.

First of all, for the definition of ConvCNPs, the translation equivariance is defined in Property 3.

**Property** **3** (Translation equivariance) [31]. *Assume* *H* *is a function space on* X*, and* *T* *and* $T^{\prime }$ *can be defined:*

$$T:\;X\times Z\to Z,\;T_{\tau }\;Z=\left(\left(x_{1}{+\tau ,\;y}_{1}\right),\cdots ,\left(x_{m}{+\tau ,\;y}_{m}\right)\right)\;T^{\prime }:\;X\times H\to H,\;T_{\tau }^{\prime }\;h\left(x\right)=h\left(x-\tau \right)$$

The mapping Φ: Z→H is called translation equivariance, if $\Phi \left(T_{\tau }\;Z\right){=T}_{\tau }^{\prime }\Phi \left(Z\right)$ for all τ∈X and Z∈Z.

**Theorem** **1.** *Assume a collection*${Z^{\prime }}_{\leq M}\subseteq Z_{\leq M}$*, which has multiplicity K. If the function*$\Phi :\;{Z^{\prime }}_{\leq M}\to C_{b}\left(X,\;Y\right)$*is permutation invariant, translation equivariant, and continuous, then*Φ*has a representation as follows* [31]:

$$\Phi \left(Z\right)=\rho \left(E\left(Z\right)\right),\;E\left(\left(x_{1}{,\;y}_{1}\right),\cdots ,\left(x_{m}{,\;y}_{m}\right)\right)=\overset{\;m}{\underset{i=1}{\sum }}\Phi \;\left(y_{i}\right)\psi \left(≔-x_{i}\right)$$

For continuous and translation-equivariant $\rho :\;H\to C_{b}\left(X,\;Y\right)$, continuous $\Phi :\;Y\to {\mathbb{R}}^{K+1}$, and ψ: X→ℝ, where H is a function space, the function Φ is called ConvDeepSet.

The key considerations of ϕ, ψ, and ρ for Φ of ConvCNPs are:

(1) Setting ψ to be a positive definite reproducing kernel Hilbert space (RKHS) [35].
(2) Setting $\phi \left(y\right){=(y}^{0}{,y}^{1},\cdots {,y}^{K})$ [36].
(3) Setting ρ to be a CNN.

The ConvCNPs can be represented by a conditional distribution, as follows:

(4) $$p\left(Y\;|\;X,\;Z\right)=\prod_{n=1}^{N}\;p\left(y_{n}{\;|\;\Phi }_{\theta }\left(Z\right)\left(x_{n}\right)\right)=\prod_{n=1}^{N}N\left(y_{n}{;\mu }_{n}{,\Sigma }_{n}\right)$$

where ${(\mu }_{n}{,\Sigma }_{n})$=${\;\Phi }_{\theta }{(Z)(x}_{n})$.

For an on-the-grid version of ρ(·), a CNN is firstly applied to *E*(*Z*), and then an MLP can map the output at each pixel in the target set to ${\mathbb{R}}^{2C}$. To summarize, the on-the-gird algorithm is given by:

(5) $$\left(\mu ,\sigma^{2}\right)=\underset{\rho }{\underbrace{\mathrm{CNN}}}\left(\overset{E(\mathrm{context}\;\mathrm{set})}{\overbrace{{\left[\underset{\mathrm{density}\;\mathrm{channel}}{\underbrace{\mathrm{Conv}\left(M_{c}\right)}};\mathrm{Conv}\left(M_{c}⊙I\right)/\underset{\mathrm{multiplies}\;\mathrm{by}\;\psi \;\mathrm{and}\;\mathrm{sums}}{\underbrace{\mathrm{Conv}\left(M_{c}\right)}}\right]}^{T}}}\right)$$

where $\left(\mu ,\sigma^{2}\right)$ are means and variances, ρ is implemented with a CNN, and *E* is produced by the mask $M_{c}$ and a convolution operation.

### 2.2. Definition of SDE-Net

Neural ordinary differential equation (ODE-Net): Neural nets such as residual networks (ResNet) [37], normalizing flows [38], and recurrent neural network decoders [39] map an input *x* to an output *y* through a sequence of hidden layers; the hidden representations can be viewed as the states of a dynamical system:

(6) $$x_{t+1}{=x}_{t}+f\;\left(x_{t},\;t\right)$$

where t ∈ {0⋯T} is the index of the layer, and $x_{t}\in {\mathbb{R}}^{D}$ is the hidden state at neural network layer *t*. The equation can be reorganized as $\frac{x_{t}+∆t-x_{t}}{∆t}={f\;(x}_{t},t)$, where ∆t=1. If we assume that ∆t→0, then we can obtain the parameterized continuous dynamics of the hidden units, which apply an ODE specified by a neural network:

(7) $$\underset{∆t\to 0}{\lim}\frac{x_{t}+∆t-x_{t}}{∆t}=\frac{{dx}_{t}}{dt}={f\;(x}_{t},\;t,\;\theta )$$

The solution of an ODE can be computed using a black-box differential equation solver to evaluate the hidden unit state wherever necessary. However, ODE-Net is a deterministic model for predictions, and cannot model epistemic uncertainty. To overcome this disadvantage, the novel SDE-Net model is proposed to characterize a stochastic dynamical system for capturing epistemic uncertainty with Brownian motion, which is widely used to model the randomness of the motion of atoms or molecules in physics.

SDE-Net: A standard Brownian motion term is added to Equation (7) to form a neural SDE dynamical system. The continuous-time dynamical system is expressed as follows:

(8) $${dx}_{t}=f\;\left(x_{t},\;t\right)dt+g\left(x_{t},\;t\right){dW}_{t}$$

where $g\left(x_{t},\;t\right)$ indicates the variance of the Brownian motion, and represents the epistemic uncertainty of the dynamical system. However, a standard Brownian motion $W_{t}$ is a stochastic process, which follows the three properties: (a) $W_{0}=0$; (b) $\nabla W=W_{t}-W_{s}$ is N(0, t−s) for all t≥s≥0; and (c) for any two different time intervals, the increments $\nabla W_{1}$ and $\nabla W_{2}$ are independent random variables.

More importantly, ${f\;(x}_{t},\;t)$ and $g\left(x_{t},\;t\right)$ in Equation (8) can be represented by NNs to construct SDE-Net. Where ${f\;(x}_{t},\;t)$ is used as the drift net to control the system in order to achieve good predictive accuracy and aleatoric uncertainty, and $g\left(x_{t},\;t\right)$ is utilized as the diffusion net to represent the epistemic uncertainty of the dynamical system. $f\;\left({x,\;t;\;\theta }_{f}\right)$ and $g\left(x_{0};{\;\theta }_{g}\right)$ must both be uniformly Lipschitz continuous. This can be satisfied by using Lipschitz nonlinear activations in the network architectures, such as ReLU, sigmoid, and Tanh.

## 3. Proposed Methods

Section 3.1 shows the architecture of vNPs–SDE net, which is implemented by different NPs for specific deep leraning tasks. Section 3.2 presents the objective function of vNPs–SDE net for uncertainty estimates. The implementation algorithms of the ConvCNPs–SDE-, ANPs–SDE-, and CNPs–SDE-Net are described in Section 3.3.

### 3.1. The Architecture of vNPs–SDE-Net

In Section 3.1.1, for synthetic 1D regression and 2D image classification tasks, we firstly apply ConvCNPs to complete the ID data with high missing rates, and then use SDE-Net to quantify the uncertainty of the noisy ID dataset.

In Section 3.1.2, for multidimensional regression tasks, we replace the downsampling NNs in SDE-Net with the encoder of CNPs or ANPs in order to encode the regression data as latent representation *r*, then use the drift net and diffusion net of SDE to deal with *r*, and finally apply the decoder of CNPs or ANPs to substitute the fully-collected NNs in SDE-Net.

#### 3.1.1. vNPs–SDE-Net for Synthetic 1D Regression and 2D Image Classification Tasks

For synthetic 1D regression and 2D image classification tasks, the latest research [31] has extensively compared the ConvCNPs model to the CNPs and ANPs models, and proved that the ConvCNPs model with translation equivariance can improve performance in off-the-grid synthetic 1D datasets or on-the-grid image datasets. The reason for the lack of comparision between vanilla NPs models and the ConvCNPs model is that the ANPs model with attention mechanisms is usually superior to vanilla NPs, which suffer a fundamental disadvantage of underfitting and give inaccurate prediction values at the observed context points on which they condition [30], so the CNPs and ANPs models can be viewed as preeminent representatives of the NPs family.

**Figure 3:**
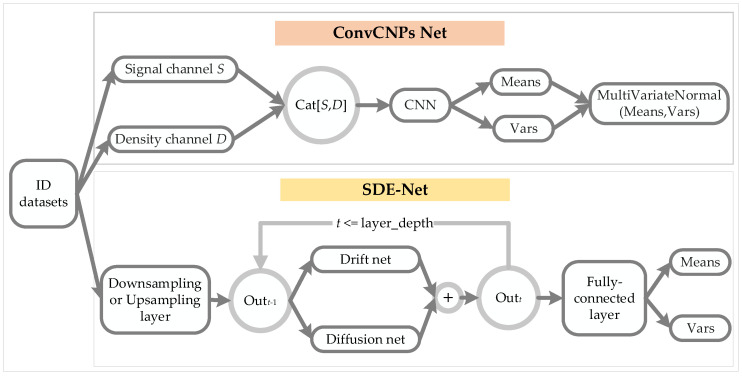
Architecture of the ConvCNPs–SDE model. ID datasets were used to train ConvCNPs-Net and SDE-Net.

In Figure 3, we introduce the architecture of the ConvCNPs–SDE model, which contains ConvCNPs and the SDE-Net model for training synthetic 1D regression and 2D image classification tasks.

For the ConvCNPs-Net model, we apply ID datasets to train ConvCNPs and SDE-Net. Where ConvCNPs select all observed context points as signal channel $S≔M_{c}⊙I$, suppose *I* stands for the image and $M_{c}$ denotes density channel $D≔M_{c}$.

We can concatenate *S* and *D* to form [*S*, *D*], which means that “there is a point at this position”. Then a convolutional neural network (CNN) is applied to a normalized [*S*, *D*] to produce Means and Vars, which can be used to generate a continuous multivariate normal distribution.

For the SDE-Net model used in 1D regression tasks, we adopt upsampling with a linear layer. The drift net uses a fully connected linear layer, and the linear layer adopts ReLU activation without batch normalization. The diffusion net can apply multiple linear layers and ReLU activations, and it has one output and employs sigmoid activation for the last layer.

For the SDE-Net model used in 2D image classification tasks, we use a downsampling layer with multiple convolutional layers for extracting features; each layer uses ReLU activation and batch normalization. The drift net consists of convolutional layers, and the input channel of each layer sets aside an extra position for layer depth, which signifies the number of discretization points of the stochastic process. The diffusion net is the same as the drift net, except for the last layer, which uses sigmoid activation function to return 0 or 1 for the diffusion net, while the drift net outputs based on the number of output channels [19].

The above mainly introduces the model structure of ConvCNPs and SDE-Net in the training phase for synthetic 1D regression and 2D classification tasks with ID datasets.

**Figure 4:**
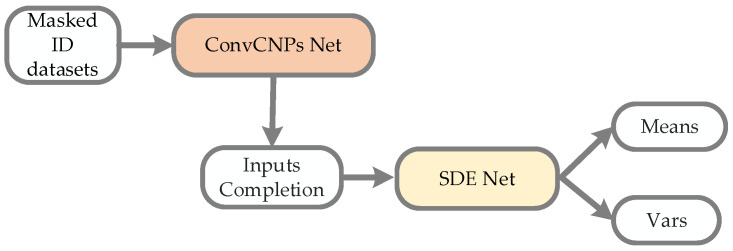
The process architecture of the ConvCNPs–SDE model for masked ID datasets. ConvCNPs-Net is used to complete the masked ID data, and then the completed data is processed with SDE-Net to produce Means and Vars.

Figure 4 shows that the ConvCNPs model can be utilized to complete masked ID datasets, and the recovered ID datasets can be more effectively and accurately recongnized by SDE-Net.

#### 3.1.2. vNPs–SDE-Net for Multidimensional Regression Tasks

For 1D regression and 2D classification tasks, the ConvCNPs model has advantages over other members of the NPs family. For multidimensional regression tasks, however, it is difficult to apply the property of translation equivariance, due to its uncertain number of dimensions, so we try to adopt ANPs and CNPs models.

For example, in the encoder of the CNPs model [29], observed context points ${\left(x,y\right)}_{1},\ldots ,{\left(x,y\right)}_{i}$ are passed through an MLP to generate representations $r_{1},\ldots ,r_{i}$, and then mean function *m* is employed to $r_{1},\ldots ,r_{i}$ to produce the global deterministic representation $r_{C}$. Specifically, the encoder in CNPs has six latent fully connected linear layers with ReLU activation, and the number of neurons in the six latent layers is equal to the dimension *n* of the regression dataset. For the decoder of the CNPs model, we still apply an MLP with five fully connected linear layers and ReLU activation; the generated representation $r_{C}$ is concatenated with the target data $x_{T}$ and together passed through the defined decoder MLP to produce the parameters Means and Vars for multivariate normal distribution.

For the encoder of the ANPs model [30], we still adopt an MLP with six fully connected linear layers to replace the self-attention mechanism for the deterministic path, and apply three fully connected linear layers for the latent path. The mean aggregation is replaced by a multihead cross-attention mechanism, and the number of heads is 10. Thus, in the deterministic path, each context point ${\left(x,y\right)}_{i}$ is passed through an MLP to produce representation $r_{i}$; the target query $x_{T}$ attends to the *i* key–value pairs $\left(x_{i}{,\;r}_{i}\right)$, and assigns weights $w_{i}$ to each pair in order to generate a representation $r^{*}=\underset{i}{\sum }w_{i}r_{i}$.

For the latent path of the ANPs model, a representation $r_{C}$ is generated in a similar manner to the encoder of the CNPs model. Global latent $r_{C}$ can be utilized to parameterise a multivariate normal distribution, which can model different realisations of the data-generating stochastic process, and sample from the distribution to produce the latent variable *z*, corresponding to one realization of the stochastic process.

For the decoder of the ANPs model, r* and *z* are concatenated with $x_{T}$ and passed through an MLP with six fully connected linear layers to produce the parameters Means and Vars of multivariate normal distribution.

**Figure 5:**
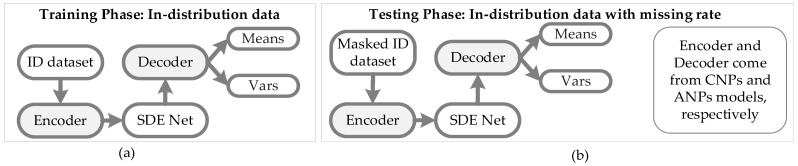
Architecture of SDE-Net with CNPs or ANPs. (**a**) An ID dataset is used to train the CNPs–SDE or ANPs–SDE models; the encoder and decoder are from the CNPs and ANPs models, respectively. (**b**) A masked ID dataset is used to test the performance of the CNPs–SDE or ANPs–SDE models.

In Figure 5a, the ID dataset is used to train the CNPs–SDE or ANPs–SDE models; the encoder and decoder are from the CNPs and ANPs models, respectively, so as to generate the parameters Means and Vars of normal distribution. In Figure 5b, a masked ID dataset is utilized to test the performance of the constructed vNPs–SDE model when facing the ID dataset with noise or a high missing rate.

The ANPs model with attention mechanisms is more expressive and accurate than the NPs and CNPs models. At test time, the computational time complexity of the NPs and CNPs models is O(n+m), because each of *n* context points passes through the MLP of the encoder to generate $r_{1},\ldots ,r_{n}$ for producing $r_{C}$, and then $r_{C}$ is incorporated with each of *m* target points in the decoder to generate *m* predicted values. However, the computational time complexity of the ANPs model increases from O(n+m) to O(n(n+m)), since the self-attention is applied to *n* contexts, and *m* target points are used to compute weights for all of *n* contexts.

### 3.2. The Objective Function of the vNPs–SDE-Net for Uncertainty Estimates

The objective function for training the vNPs–SDE-Net model is:

(9) $$\underset{\theta_{\mathrm{vNP}}}{\min}E_{x_{0}\sim P_{\mathrm{train}}}E\left(\mathrm{LogP}\left(x_{0}\right)\right)+\underset{\theta_{f}}{\min}E_{x_{0}\sim P_{\mathrm{train}}}E\left(L\left(x_{T}\right)\right)\;+\underset{\theta_{g}}{\min}E_{x_{0}\sim P_{\mathrm{train}}}g\left(x_{0};\theta_{g}\right)+\underset{\theta_{g}}{\min}E_{{\tilde{x}}_{0}\sim P_{\mathrm{OOD}}}g\left({\tilde{x}}_{0};\theta_{g}\right)\;s.t.\;{dx}_{t}=\underset{\mathrm{drift}\;\mathrm{neural}\;\mathrm{net}}{\underbrace{f\;\left(x_{t},\;t;\theta_{f}\right)}}\;dt+\underset{\mathrm{diffusion}\;\mathrm{neural}\;\mathrm{net}}{\underbrace{g\left(x_{0};\theta_{g}\right)}}{dW}_{t}$$

where LogP(·) in Equation (9) is the log-likelihood loss function as a reconstruction term for the ConvCNPs and CNPs models. However, for the ANPs model, the first item of the objective function in Equation (9) is the evidence lower bound (ELBO), which includes a reconstruction term and a Kullback–Leibler divergence (KL) term. This is because the ANPs model has a latent path through which to generate latent variable *z* for modelling uncertainty. L(·) is the loss function dependent on the task, such as cross-entropy loss for classification tasks and log-likelihood loss for regression tasks; *T* is the terminal time of the stochastic processes; $P_{\mathrm{train}}$ is the distribution of the training data; and $P_{\mathrm{OOD}}$ represents the OOD data. The OOD data can be obtained by adding additive Gaussian noise to the noisy inputs ${\tilde{x}}_{0}=x_{0}+\epsilon$, and then distributing the inputs according to the convolved distribution.

Once an vNPs–SDE-Net has been trained, we can obtain multiple random realizations of the SDE-Net in order to get samples ${\left\{x_{T}\right\}}_{m=1}^{M}$, and then compute the two uncertainties from them. The aleatoric uncertainty is given by the expected predictive entropy $E_{p\left(x_{T}{|x}_{0}{,\;\theta }_{f,g}\right)}\left[ℋ\left[p\left({y|x}_{T}\right)\right]\right]$ in classification, and by the expected predictive variance $E_{p\left(x_{T}{|x}_{0}{,\;\theta }_{f,g}\right)}\left[\sigma \left(x_{T}\right)\right]$ in regression. The epistemic uncertainty is given by the variance of the final solution $\mathrm{Var}\left(x_{T}\right)$.

After the vNPs and SDE-Net models are trained, suppose that the ID dataset *I* with missing rate MR and mask = Bernoulli (1-MR), so the masked ID dataset *I* can be expressed as mask∗I. The test performance of vNPs–SDE for the masked ID dataset can be processed as follows:

(1) Completed_*I* = vNPs (mask∗I)
(2) Means, Vars = SDE-Net(Completed_*I*)

Since our purpose is to perform supervised learning and uncertainty quantification, the simple Euler–Maruyama method with fixed step size is adopted for model training. Hence, the time interval [0, *T*] is divided into *N* subintervals, and SDE can be simulated as:

(10) $$x_{k+1}=x_{k}+f\left(x_{k}{,\;t;\theta }_{f}\right)∆t+g\left(x_{0}{;\theta }_{g}\right)\sqrt{∆t}{\;Z}_{k}$$

where $Z_{k}\sim N\left(0,1\right)$ and ∆t=T/N. The number of steps for solving the SDE can be regarded equivalently as the number of layers in the NNs. Moreover, the training of SDE-Net is actually the same as in NNs. The vNPs–SDE model is optimized in the Algorithms in the following sections.

### 3.3. The Implementation of vNPs–SDE-Net

In this section, vNPs are implemented with ConvCNPs for 1D regression and 2D image classification tasks in Algorithm 1, and vNPs are realized with CNPs and ANPs for multidimensional regression tasks in Algorithm 2.

#### 3.3.1. The Implementation of vNPs–SDE-Net with ConvCNPs

Assume an ID dataset ${\left\{{\left(x,y\right)}_{i}\right\}}_{i=1}^{n}\sim \;P\left(x,y\right)$ for 1D regression or 2D image classification tasks, and then sample a minibatch of *m* data, which includes inputs $X^{m}$ and targets $Y^{m}$ from the ID dataset: $\left(X^{m},Y^{m}\right){\;\sim \;p}_{\mathrm{ID}}\left(x,y\right)$.

For training the ConvCNPs model, suppose that the context rate (*CR*) is 80%, so the context dataset $\left(X_{c}^{m},{\;Y}_{c}^{m}\right)$ is composed of 80% of *m* datasets, and the sampled *m* data are viewed as the target dataset $\left(X_{t}^{m},{\;Y}_{t}^{m}\right)$. The output of the ConvCNPs model is a multivariate normal distribution, so the predicted Means and Vars can be called by the ConvCNPs model, the purpose of which is to complete the masked ID dataset.

For the SDE-Net model, the purpose of the training of the drift net is to fit the ID dataset. Meanwhile, for the training diffusion net *g*, the dataset from ID or OOD is respectively marked with labels 0 and 1, and the purpose of *g* is to distinguish whether the dataset comes from ID or OOD, so the purpose of training *g* is to minimize or maximize the binary cross-entropy loss function for the ID dataset or the OOD dataset, respectively.
**Algorithm 1** Implementation of the ConvCNPs–SDE model**Inputs:** ID dataset $p_{\mathrm{ID}}\left(x,y\right)$; *CR* and MR are the context rate and missing rate, respectively; *ccnps* represents the ConvCNPs model of vNPs for completing the ID dataset; $h_{1}$ is the downsampling net for 2D image classification tasks or the upsampling net for 1D regression tasks; $h_{2}$ is the fully connected net; *f* represents the drift net and *g* represents the diffusion net; *t* is the layer depth; $L_{1}$ is the cross-entropy loss function, $L_{2}$ is the log-likelihood loss function, and $L_{3}$ is the binary cross-entropy loss function. **Outputs**: Means and Vars **for** #training iterations **do**1. Sample a minibatch of *m* data: $\left(X^{m},Y^{m}\right){\;\sim \;p}_{\mathrm{ID}}\left(x,y\right)$;2. **if** for 1D regression task:3. Context points $\left(X_{c}^{m},Y_{c}^{m}\right)$ are generated from sampled target points $\left(X_{t}^{m},Y_{t}^{m}\right)$ based on *CR*, where $\left(X_{t}^{m},Y_{t}^{m}\right)$ equals $\left(X^{m},Y^{m}\right)$;4. Forward through the ConvCNPs model: *Y_dist* = *ccnps*$\left(X_{c}^{m},Y_{c}^{m},X_{t}^{m}\right)$;5. Forward through the upsampling net of the SDE-Net block: $X_{0}{}^{m}=h_{1}\left(X^{m},Y^{m}\right)$;6. **else** for 2D image classification task:7. Forward through the ConvCNPs model: *Y_dist* = *ccnps*$\left(X^{m},Y^{m}\right)$;8. Forward through the downsampling net of the SDE-Net block: $X_{0}{}^{m}=h_{1}\left(X^{m},Y^{m}\right)$;9. **for** *k* = 0 to *t* − 1 **do**10. Sample $Z_{k}{}^{m}\sim N\left(0,1\right)$; 
$X_{k+1}{}^{m}=X_{k}{}^{m}+f\;\left(X_{k}{}^{m},t\right)∆t+g\left(X_{0}{}^{m}\right)\sqrt{∆t}Z_{k}$;11. **end for**12. Forward through the fully connected layer of the SDE-Net block: $Y_{f}{}^{m}=h_{2}\left(X_{k}{}^{m}\right)$;13. Update $h_{1}{,\;h}_{2}$ and *f* by $\nabla_{{\;h}_{1}{,\;h}_{2}}\;\mathrm{and}\;f\;\frac{1}{m}L_{1}\left(Y_{f}{}^{m},Y^{m}\right)$;14. Update *ccnps* by $\nabla_{\mathrm{ccnps}\;}\frac{1}{m}L_{2}\left(Y\_\mathrm{dist}.\mathrm{means},Y_{t}^{m}\right)$;15. Sample a minibatch of m data from ID: $\left(X^{m},0\right)\sim p_{\mathrm{ID}}\left(x,y\right)$;16. Sample a minibatch of m data from OOD: $\left({\tilde{X}}^{m},1)\sim p_{\mathrm{OOD}}(x,y\right)$;17. Forward through the downsampling or upsampling nets of the SDE-Net block:
${X_{0}}^{m},{{\tilde{X}}_{0}}^{m}=h_{1}(X^{m}),h_{1}({\tilde{X}}^{m})$;18. Update *g* by $\nabla_{g}\frac{1}{m}L_{3}\left(g\left({X_{0}}^{m}\right),0\right)-\nabla_{g}L_{3}\left(g\left({{\tilde{X}}_{0}}^{m}\right),1\right)$;**for** #testing iterations **do**19. Evaluate the of ConvCNPs–SDE model;20. Sample a minibatch of *m* data from ID: $\left(X^{m},Y^{m}\right)\sim {\;p}_{\mathrm{ID}}\left(x,y\right)$;21. *mask* = Bernoulli (1-MR)22. *masked_*$X^{m}$ = *mask* ∗ $X^{m}$;23. *completed_*$X^{m}$= *ccnps*$\left({\mathrm{masked}\_X}^{m}\right)$;24. Means, Vars = SDE-Net(*completed_*$X^{m}$);


For 1D regression tasks, the specific settings of SDE-Net include a layer depth of four; the upsampling net has a linear layer with a [1–50] architecture, the drift net has a fully connected linear layer with a [50–50] architecture, and the diffusion net has a [50–100–100–1] fully connected linear layer. ReLU is the activation function. The number of training epochs is 1000 and the optimizer is an SGD. The learning rate for the diffusion net is 0.01, while for the drift net it is 1e-4, and the learning rate is multiplied by 0.1 when the number of epochs reaches 60 and the momentum and weight decay are 0.9 and 5e-4, respectively. We reshape the generated ID dataset to (batch_size, total _points), where batch_size = 15 and total_points = 100. Assume the MR is given, so the remaining training dataset is (1-MR) × total_points for the 1D tasks.

For the 1D regression tasks, the specific settings of the ConvCNPs–SDE model include the ConvCNPs net having four 1D convolution layers, which can be described as [in_channel, out_channel, kernel_size, and stride, padding], and specifically contain {[3, 16, 5, 1, 2], [16, 32, 5, 1, 2], [32, 16, 5, 1, 2], and [16, 2, 5, 1, 2]}. The density is 25 and *K* = 1 for $\phi \left(y\right)=\left({\;y}^{0}{,\;y}^{1}{,\ldots ,\;y}^{K}\right)$, and the RBF is chosen for covariance ψ(y); thus, these settings can satisfiy Theorem 1. The model settings of SDE-Net are the same as those defined in the previous paragraph, except for the upsampling layer, which concatenates *x* and *y* as inputs and has fully connected linear NNs with a [2–50] architecture. ReLU is the activation function. For training ConvCNPs–Net, the number of training epochs is 1000, Adam is the optimizer, and the learning rate and weight decay are 1e-3 and 1e-5, respectively. The setting of the training parameters is the same as for SDE-Net in the previous paragraph.

For the 2D image classification tasks, vanilla SDE-Net follows the settings of [19]. For the convolutional layer, the downsampling layers contain three 2D convolution (Conv2d) layers, which can be described as {[1, 64, 3, 1, 0], [64, 64, 4, 2, 1], and [64, 64, 4, 2, 1]} for the MNIST dataset and {[3, 64, 3, 1, 0], [64, 64, 4, 2, 1], and [64, 64, 4, 2, 1]} for the CIFAR10 dataset. The drift net contains two Conv2d layers with {[65, 64, 3, 1, 1], and [65, 64, 3, 1, 1]}, and the diffusion net has the same convolution layers as the drift net, but the diffusion net owns an extra linear connection [64–1] in the last layer. The fully connected layer of SDE-Net is [64–10]. In order to train the SDE-Net, the layer depth is 6 and the number of training epochs is 40, An SGD is used as the optimizer, the learning rates of diffusion net are 0.01 and 0.005 for MNIST and CIAFAR10, respectively, and the learning rates of the other nets are 0.1. The momentum and weight decay are 0.9 and 5e-4, respectively.

For the 2D image classification tasks, the ConvCNPs-Net of the ConvCNPs–SDE model selects all observed context points as signal channel $S≔M_{c}⊙\mathrm{Inputs}$, assuming *Inputs* stands for the image and $M_{c}$ denotes density channel $D≔M_{c}$, where $M_{c}=\mathrm{Bernoulli}(\frac{\mathrm{number}\;\mathrm{of}\;\mathrm{context}\;\mathrm{pixel}\;\mathrm{points}}{\mathrm{number}\;\mathrm{of}\;\mathrm{total}\;\mathrm{pixel}\;\mathrm{points}})$ and the number of context points is uniformly sampled from $\left[\frac{\mathrm{number}\;\mathrm{of}\;\mathrm{total}\;\mathrm{pixel}\;\mathrm{points}}{100},\;\frac{\mathrm{number}\;\mathrm{of}\;\mathrm{total}\;\mathrm{pixel}\;\mathrm{points}}{2}\right]$. *S* and *D* are firstly processed by a Conv2d layer {[1, 64, 9, 1, 4]} for the MNIST dataset and {[3, 64, 9, 1, 4]} for the CIFAR10 dataset in order to generate *S*’ and *D*’, and then we can concatenate them to form [*S*’, *D*’], which is passed through a CNN, and finally the output of the CNN is transformed to a continuous function space for translation equivariance. The CNN firstly has a Conv2d layer with {[128, 64, 1, 1, 0]}, and then has eight Conv2d residual blocks—each residual block having two Conv2d layers with {[64, 64, 5, 1, 2]}—and finally owns the last Conv2d layer with {[64, 2, 1, 1, 0]} for the MNIST dataset and {[64, 6, 1, 1, 0]} for the CIFAR10 dataset. The training parameters include 20 training epochs; Adam is chosen as the optimizer, the batch size is 16, and the learning rate is 5e-4. The settings of SDE-Net in the ConvCNPs–SDE model are the same as in the previous paragraph.

#### 3.3.2. The Implementation of vNPs–SDE-Net with CNPs or ANPs

For the multidimensional regression tasks, we apply vanilla CNPs or ANPs to represent vNPs. As described in Figure 5, the downsampling net and the fully connected layer are replaced by the encoder and decoder of the CNPs or ANPs models. The training and testing processes are described in Algorithm 2:
**Algorithm 2** Implementation of the CNPs–SDE or ANPs–SDE models**Inputs**: ID dataset $p_{\mathrm{ID}}\left(x,y\right)$; MR is the missing rate of the ID dataset; the downsampling layer $h_{1}$ is the encoder of the CNPs or ANPs models; *f* and *g* are the drift net and diffusion net, respectively;${\;L}_{1}$ is the negative log-likelihood loss function for the CNPs model or the ELBO for the ANPs model; $L_{2}$ is the binary cross-entropy loss function; the fully collected layer $h_{2}$ is the decoder of the CNPs or ANPs models to produce Means and Vars. **Outputs**: Means and Vars **for** #training iterations **do**1. Sample a minibatch of *m* data: $\left(X^{m},Y^{m}\right){\;\sim \;p}_{\mathrm{ID}}\left(x,y\right)$;2. Forward through the downsampling net: *d_mean_z* = $h_{1}\left(X^{m}\right)$ and $X_{0}{}^{m}=\left(X^{m},\;d\_\mathrm{mean}\_z\;\right)$;3. Forward through the SDE-Net block:4. **for** *k* = 0 to n−1
**do**5. Sample $Z_{k}{}^{{\;N}_{M}}\sim N\;\left(0,1\right)$;6. $X_{k+1}{}^{m}=X_{k}{}^{m}+f\;\left(X_{0}{}^{m},t\right)∆t+g\left(X_{0}{}^{m}\right)\sqrt{∆t}Z_{k}$;7. **end for**8. Means, Vars = $h_{2}$($X_{k+1}{}^{m}$)9. Update $h_{1}{,\;h}_{2}$ and *f* by $\nabla_{{\;h}_{1}{,\;h}_{2}}\;\mathrm{and}\;f\;\frac{1}{m}L_{1}\left(\mathrm{Means},Y^{m}\right)$;10. Sample a minibatch of m data from ID: $\left(X^{m},0\right)\sim p_{\mathrm{ID}}\left(x,y\right)$;11. Sample a minibatch of $\left({\tilde{X}}^{m},1)\sim p_{\mathrm{OOD}}(x,y\right)$;12. Forward through the downsampling or upsampling nets of the SDE-Net block: ${X_{0}}^{m},{{\tilde{X}}_{0}}^{m}=h_{1}(X^{m}),h_{1}({\tilde{X}}^{m})$;13. Update *g* by $\nabla_{g}\frac{1}{m}L_{2}\left(g\left({X_{0}}^{m}\right),0\right)-\nabla_{g}L_{2}\left(g\left({{\tilde{X}}_{0}}^{m}\right),1\right)$;**for** #testing iterations **do**14. Evaluate the CNPs–SDE or ANPs–SDE models;15. Sample a minibatch of *m* data from ID: $\left(X^{m}{,Y}^{m}\right)\sim p_{\mathrm{ID}}\left(x,y\right)$;16. *mask* = Bernoulli(1-MR);17. *masked_*$X^{m}$ = *mask* ∗ $X^{m}$;18. Means, Vars = CNPs_SDE (*masked_*$X^{m}$) or ANPs_SDE (*masked_*$X^{m}$).

As the dimension of YearPredictionMSD is 90, we apply a fully connected DNN with a [91–90–90–90–90–90] architecture for the encoder of the CNPs model, and the decoder architecture of the CNPs model is [180–90–90–90–2]. For SDE-Net, the drift net has a fully connected linear layer with a [180–180] architecture, while the diffusion net has a [180–100–1] DNN architecture, and the layer depth is four. ReLU is the activation function. The number of training epochs is 60 and the optimizer is an SGD; the learning rate for the diffusion net is 0.01, while for the drift net it is 1e-4, and the learning rate is multiplied by 0.1 when the number of epochs reaches 30 and the momentum and weight decay are 0.9 and 5e-4, respectively.

For the ANPs of the ANPs–SDE model, the deterministic path and latent path of the encoder have fully connected DNN architecture—[91–90–90–90–90–90] and [91–90–90–180], respectively. The cross-attention encoder of (keys, queries) has linear layers with a [90–90] architecture, and a multihead attention mechanism is adopted in order to deal with the processed (keys, queries), where the embedded dimension is 90 and the number of parallel attention heads is 10. The decoder of the ANPs has a fully connected DNN with a [270–90–90–90–2] architecture. For vanilla SDE-Net, the architecture of the drift net is [270–270], while that of the diffusion net is [270–100–1], and the layer depth is four. ReLU is the activation function. The training parameter settings of the ANPs–SDE model are the same as those of the CNPs–SDE model.

## 4. Results

Section 4.1 introduces the evaluation metrics. Section 4.2 presents the specific settings of the vNPs–SDE model and the expermental results.

### 4.1. Evaluation Metrics

For an OOD detection task under both classification and regression settings, assume *P* represents real postive, *N* stands for real negative, *T* indicates true prediction, and *F* signifies false prediction. Thus, *TP* implies that predicted samples and real samples are postive, *FP* denotes that predicted samples are positive and real samples are negative, *FN* betokens that predicted samples are postive and real samples are positive, and *TN* symbolizes that predicted samples and real samples are negative.

We follow previous works such as [19,23], and apply several metrics [40] for the OOD detection task. Larger values of these metrics indicate better detection performance. *N* represents that the number of TPR ∈ [0.94, 0.96].

(11) $$\mathrm{TPR}=\frac{\mathrm{TP}}{\mathrm{TP}+\mathrm{FN}}$$

(12) $$\mathrm{FPR}=\frac{\mathrm{FP}}{\mathrm{FP}+\mathrm{TN}}$$

(13) $$\mathrm{TNR}\;\mathrm{at}\;95\%\;\mathrm{TPR}=1-\frac{\mathrm{FPR}}{N}$$

(14) $$\mathrm{Precision}=\frac{\mathrm{TP}}{\mathrm{TP}+\mathrm{FP}}$$

(15) $$\mathrm{Recall}=\frac{\mathrm{TP}}{\mathrm{TP}+\mathrm{FN}}$$

(16) $$A\mathrm{ccuracy}=\frac{\mathrm{TP}+\mathrm{TN}}{\mathrm{TP}+\mathrm{TN}+\mathrm{FP}+\mathrm{FN}}$$

*AUROC* is used to denote the area under the receiver operating characteristic (*ROC*) curve; the horizontal axis of the *ROC* curve is represented by *FPR,* and the vertical axis is represented by *TPR*, so the points (*FPR*, *TPR*) on the *ROC* curve are coordinate points in the 2D Cartesian coordinate system. The probability of predicting the positive sample as postive is *p*1, and that of predicting the negative sample as positive is *p*2. Therefore, *AUROC* reflects the sorting ability of the classifier on the samples. In addition, *AUROC* is not sensitive to whether or not the sample categories are balanced, which is also the reason that *AUROC* is usually used to evaluate the classification performance of unbalanced samples.

(17) $$\mathrm{AUROC}=\frac{\sum_{i\in P}r_{i}-\frac{\left|P\right|\times \left(\left|P\right|+1\right)}{2}}{\left|P\right|\times \left|N\right|}$$

where *P* denotes a positive sample set, *N* signifies a negative sample set, | · | stands for the number of samples, and $r_{i}$ respresents the rank of element *i* in the total set (*P* + *N*), from the smallest to the largest, according to the predicted scores.

*AUPR* is used to represent fthe area under the precision-recall (*PR*) curve; the horizontal axis of the *PR* curve is represented by *Recall*, while the vertical axis is represented by *Precision*.

### 4.2. vNPs–SDE Model for ID Dataset with MR

In this section we present the experimental results of the vNPs–SDE model for the ID dataset with MR. Specifically, the ConvCNPs-Net is performed for the synthetic 1D regression task in Section 4.2.1, the vNPs–SDE model is implemented with CNPs and ANPs for the multidimensional regression task in Section 4.2.2, and the ConvCNPs–SDE model is performed with the benchmark datasets MNIST and CIFAR10 in Section 4.2.3 and Section 4.2.4, respectively.

We compare our vNPs–SDE model with the following methods for uncertainty estimates: (1) SDE-Net, which is the lastest non-Bayesian approach for uncertainty estimation in DNNs; (2) the approximate Bayesian method MC-dropout; and (3) the Bayesian approach BBP.

#### 4.2.1. ConvCNPs–SDE-Net for Synthetic 1D Regression Tasks

In order to obtain the synthetic 1D dataset, the radial basis function (RBF) kernel was used for GPs to generate the synthetic 1D dataset [29,31]. The RBF kernel was $k(x,x^{\prime })=\sigma_{1}*e^{-\frac{1}{2\sigma_{2}}{\left(x-x^{\prime }\right)}^{2}}$, and we set the output scale to $\sigma_{1}=0.5$ and the length scale to $\sigma_{2}=0.5$. We sampled 1500 *x*-axis data points from the *domain* [−2, 2] based on uniform distribution, and then the covariance of the GPs could be obtained from the sampled data points applied to the RBF, and the offset was 1e-5. If the means of the GPs are zeros and the GPs have noise—which has a mean of zero and standard deviation of 0.02—then we can generate 1500 *y*-axis GP data points for each batch. For the reproducibility of the experiment, the seed was 123.

In order to train SDE-Net, BBP, and ConvCNPs–SDE-Net, we firstly sorted the sampled *x*-axis data points from the smallest to largest, and then adjusted the generated *y*-axis data points corresponding to the *x*-axis to form the training GPs dataset ${\left\{{\left(x,y\right)}_{i}\right\}}_{i=1}^{1500}$.

**Figure 6:**
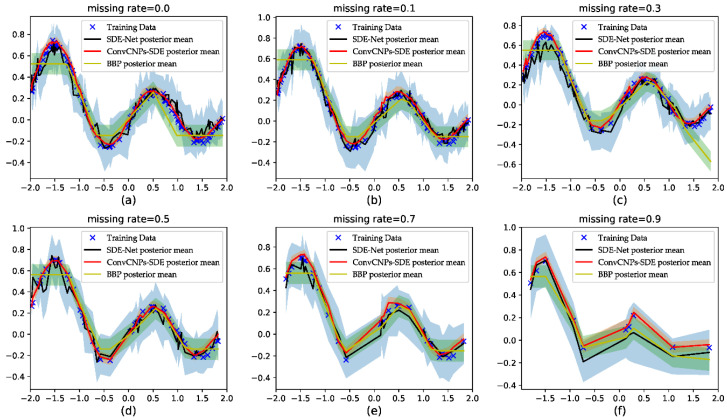
Visualizing the uncertainty of predictive distribution in regression tasks. (**a**–**f**) show that (1-MR) × 100 training data with MR = [0.0, 0.1, 0.3, 0.5, 0.7, 0.9], respectively, is used to train the SDE-Net, BBP, and the proposed ConvCNPs–SDE-Net.

The results are shown in Figure 6. The blue cross stands for training data, the black line represents the SDE-Net’s posterior mean, the red line denotes the ConvCNPs–SDE-net’s posterior mean, the yellow represents the BBP approach’s posterior mean, and the shaded region signifies the mean ± 3 standard deviations. Since MC-Dropout produced poor results, it is not shown in Figure 6. Firstly, we find that as the MR increases, the ConvCNPs–SDE model can fit the remaining data points more accurately than vanilla SDE-Net and the BBP model. Secondly, the ConvCNPs–SDE model has smaller variance than the SDE-Net and BBP models. Lastly, and most importantly, the curve of the ConvCNPs–SDE model is smoother than that of the vanilla SDE-Net and BBP models in almost all experiments, which is significant for those who have not sampled continuous *x*-axis samples in domain [−2, 2] to predict corresponding *y* values.

#### 4.2.2. The CNPs–SDE and ANPs–SDE Models for Multidimensional Regression Tasks

We followed the methods of [19], applying the YearPredictionMSD multidimensional regression dataset for this experiment. The YearPredictionMSD dataset is used to predict the release year of a song from audio features [41], and the year ranges from 1922 to 2011. The dataset has 515,345 instances and 90 attributes. We followed the train/test split—the first 463,715 examples for training and the last 51,630 examples for testing.

We obtained randomized *mask* = Bernoulli (1-MR), where MR was chosen from [0.1, 0.3, 0.5, 0.7, 0.9] to compute the RMSE. The masked YearPredictionMSD dataset can be expressed as mask∗YearPredictionMSD. The BBP, MC-dropput, ANPs–SDE-Net, SDE-Net, and CNPs–SDE-Net models were run independently six times, and the results of the RMSE are shown in Table 1. Firstly, we can conclude that as the MR increases, the RMSE values gradually increase. Secondly, the CNPs–SDE model is more accurate than the BBP, MC-dropout, ANPs–SDE and SDE-Net models.

We applied the YearPredictionMSD dataset as ID data and the Boston Housing dataset as test OOD data. Table 2 shows the OOD detection performance for different models.

We report the average performance and standard deviation for five random initializations in Table 2. Because of the imbance of the test ID and OOD datasets, AUPR out is a better metric than AUPR in [19], so ANPs–SDE-Net is a more effective method than the other methods with respect to *AUPR in* and *AUPR out*, and the conclusions drawn from other metrics show that ANPs–SDE-Net is also comparable or superior to BBP, MC-dropout, and SDE-Net.

The experimental results of Table 1 and Table 2 show that vNPs can not only help SDE-Net improve the accuracy of the ID dataset with MR, but also improve the OOD detection performance.

#### 4.2.3. ConvCNPs–SDE-Net for Image Classification Dataset: MNIST

**Figure 7:**
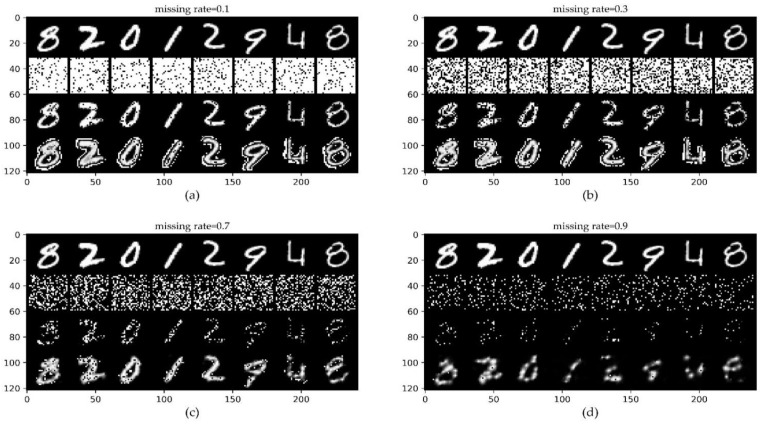
ConvCNPs-Net for MNIST with MR. (**a**–**d**) show that the missing rate takes values from [0.1, 0.3, 0.7, 0.9], respectively, to test the performance of the trained ConvCNPs-Net and ConvCNPs–SDE models. For each panel, the top row shows the original ID data MNIST, the second row demonstrates the mask based on MR, the third row exhibits the ID MNIST data with the mask, and the bottom row displays how the masked MNIST data are completed by the ConvCNPs of the ConvCNP–SDE model.

The benchmark MNIST dataset is a dataset of handwritten digits from 0 to 9, which consists of 70,000 28×28 monochrome images, including 60,000 training images and 10,000 test images [38]. The results of Figure 7 show that even when 70% of the original MNIST dataset is lost, we can roughly distinguish the values of the completed digits with the naked eye.

The drift net in SDE-Net can precisely fit the ID dataset MNIST for classification, and the diffusion net of SDE-Net directly models the relationship between the ID dataset MNIST and epistemic uncertainty; this idea encourages SDE-Net to output greater uncertainty for OOD dataset SVHH and low uncertainty for ID dataset MNIST. However, SDE-Net needs to improve the test performance for receiving the ID dataset MNIST with MR. Thus, we evaluated the performance of the BBP, MC-dropout, ConvCNPs–SDE, and SDE-Net models for OOD detection and ID with MR in classification tasks. MR takes values from [0.1, 0.3, 0.5, 0.7, 0.9, RMR], where RMR denotes random sampling from [0.5, 0.7, 0.9] each time. We report the average performance and standard deviation for five random initializations.

Table 3 shows that as the MR increases, the ConvCNPs–SDE model gradually exceeds the vanilla SDE-Net, BBP, and MC-dropout models in all metrics, including the MR obtained by random sampling.

Aside from OOD data detection, it is also significant that the application of uncertainty makes the model aware of the possibility of making mistakes in test time. Hence, the misclassification detection aims at exploiting the predictive uncertainty to distinguish the test dataset with MR in which the model has misclassified [19,30]. Table 4 shows the misclassification detection performance of the BBP, MC-dropout, ConvCNPs–SDE, and SDE-Net models on ID dataset MNIST with MR = [0.1, 0.3, 0.5, 0.7, 0.9, RMR] and SVHN. We report the average performance and standard deviation for five random initializations.

Table 4 shows that as the MR increases, the ConvCNPs–SDE model consistently surpasses the BBP, MC-dropout, and vanilla SDE-Net models in the first four metrics, including the MR obtained by random sampling. Possibly as a result of the imbalance of the test ID and OOD datasets, BBP achieves comparable or even better performance compared to the MC-dropout, ConvCNPs–SDE, and SDE-Net models in the last metric *AUPR err*. Overall, the ConvCNPs–SDE model is a better model for misclassification tasks in practice.

#### 4.2.4. ConvCNPs–SDE-Net for Image Classification Dataset: CIFAR10

The benchmark dataset CIFAR10 has 60,000 color images with 10 classes, including 50,000 training images and 10,000 test images; each class has 6000 images, and the pixel of each image is 3×32×32 [39].

**Figure 8:**
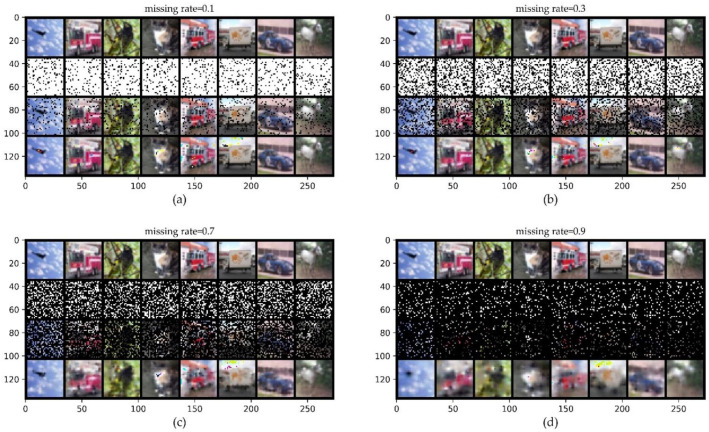
ConvCNPs-Net for CIFAR10 with MR. (**a**–**d**) show that the missing rate takes values from [0.1, 0.3, 0.7, 0.9], respectively, to test the performance of the trained ConvCNPs-Net and ConvCNP–SDE models. For each panel, the top row shows the original ID data of CIFAR10, the second row demonstrates the mask based on MR, the third row exhibits the CIFAR10 data with the mask, and the bottom row displays how the masked CIFAR10 data are completed by the ConvCNPs of the ConvCNP–SDE model.

Figure 8 shows that even when 70% of the original CIFAR10 is lost, we can generally distinguish the objects in the completed images with the naked eye in the bottom row. We evaluate the performance of the ConvCNPs–SDE and SDE-Net models for OOD dataset SVHN detection and ID dataset CIFAR10 with MR in classification tasks. We report the average performance and standard deviation for five random initializations.

Table 5 shows that the ConvCNPs–SDE model exceeds the other models in the important classification accuracy metric, while BBP surpasses almost all of the other models in the remaining four metrics for all MR values, which shows that the ability of BBP to identify SVHN as OOD data is better than that of the other methods. From the perspective of classification accuracy, the ConvCNPs–SDE model is a better model for OOD detection with benchmark dataset CIFAR10 in practice.

Table 6 shows the misclassification detection performance of the BBP, MC-dropout, ConvCNPs–SDE, and SDE-Net models on ID dataset CIFAR10 with MR and SVHN. We report the average performance and standard deviation for five random initializations. For the different values of MR, the ConvCNPs–SDE model surpasses the BBP, MC-dropout, and vanilla SDE-Net models in the first four metrics. Possibly as a result of the imbalance of the test ID and OOD datasets, SDE-Net achieves better performance than the other models in the last metric *AUPR err*. Overall, the ConvCNPs–SDE model is a better model for misclassification tasks in practice for CIFAR10.

## 5. Discussion

In this work, we proposed to incorporate the NPs family into SDE-Net to form a vNPs–SDE model for handling noisy ID datasets. The vNPs–SDE model was implemented with ConvCNPs-Net for synthetic 1D regression and 2D image classification tasks, and the vNPs–SDE model was implemented with CNPs and ANPs for multidimensional regression tasks.

For the multidimension regression tasks, the results of five models including BBP, MC-dropout, SDE-Net, CNPs–SDE, and ANPs–SDE are demonstrated in Table 1 and Table 2. Table 1 shows that as the MR increases, the values of RMSE gradually increase, and the CNPs–SDE model is more accurate than the other models. Table 2 shows that SDE-Net still has the optimal performance in RMSE, TNR at TPR 95%, and detection accuracy. Additionally, compared to the other models, SDE-Net has the fewest parameters. However, ANPs–SDE-Net is a more effective method than the other methods in terms of *AUROC*, *AUPR in*, and *AUPR out*, and is comparable to SDE-Net in RMSE.

The results of the three models—namely, BBP, SDE-Net, and ConvCNPs–SDE—are plotted in Figure 6 for synthetic 1D data. Due to MC-dropout method not fitting the GPs dataset, the results of MC-dropout are not shown in Figure 6. More specifically, our proposed ConvCNPs–SDE model can fit the synthetic 1D data better and produce smaller variances than the BBP and SDE-Net models. When MR is 0.9, we find that the results of the BBP and SDE-Net models deviate from the data. At the beginning and the end of the training set, BBP produces worse results than SDE-Net and ConvCNPs–SDE; this may be due to the learning Bayesian phase at the beginning and the uncertainty introduced about unseen data [8].

The results of the four models, including BBP, MC-dropout, SDE-Net, and ConvCNPs–SDE, are given in Table 3 and Table 4 for MNIST. Table 3 shows that as the MR increases, the ConvCNPs–SDE model gradually surpasses the vanilla SDE-Net, BBP, and MC-dropout models in all metrics, including the MR obtained by random sampling; this indicates that even with noisy ID data, our proposed ConvCNPs–SDE model can still effectively detect OOD data. Table 4 describes the misclassification detection when exploiting the proposed ConvCNPs–SDE model to distinguish between the dataset with MR and the OOD data. Table 4 shows that as the MR increases, the ConvCNPs–SDE model consistently surpasses the BBP, MC-dropout, and vanilla SDE-Net models in the first four metrics. However, BBP achieves comparable or better performance compared to the MC-dropout, ConvCNPs–SDE, and SDE-Net modesl in the last metric *AUPR err;* this may be as a result of the imbalance of the ID and OOD data [19], or due to noisy ID data, and as such deserves further study.

For the benchmark CIFAR10 dataset, the results are depicted in Table 5 and Table 6. Table 5 shows that the ConvCNPs–SDE model is superior to the other methods in terms of classification accuracy, while BBP surpasses almost all of the other models in the remaining four metrics for all ID data with MR values, which means that the ability of BBP to identify SVHN as OOD data is better than that of the other methods. Table 3 and Table 5 show the OOD detection for MNIST and CIAFR10, respectively, but we have different conclusions for distinguishing between OOD and noisy ID data. We can assume that the BBP method, as illustrated in Table 5, has some advantages over the other approaches in terms of OOD detection, with three large input channels. For the ID dataset, the limitation of our proposed ConvCNPs–SDE model may be that the ordinary DNNs in the ConvCNPs–SDE model cause poor performance. Table 4 and Table 6 have smiliar conclusions.

Despite these promising results, future studies should train and test our models on more datasets in order to verify their performance, and at the same time test the OOD detection perforamnce of BBP on other noisy ID datasets. Moreover, the local optimal characteristics of DNNs lead to instability of the predicted results; as such, future research should explore other methods to make the loss values of DNNs close to a constant.

## 6. Conclusions

SDE-Net is a much simpler and more straightforward method than BNNs for uncertainty estimates in deep neural networks, and it can separate different sources of uncertainties and accurately distinguish between ID and OOD datasets. It is a promising method for equipping NNs with meaningful uncertainties in many safety-critical fields, such as medical diagnoses and self-driving vehicles. However, SDE-Net does not consider the general situation in a wider field—for instance, ID data with noise or high missing rates in practice.

In this paper, we proposed a vNPs–SDE model, which combines SDE-Net with the NPs family in order to deal with the noisy ID dataset for uncertainty estimates. Specifically, we applied the permutation invariance property of CNPs and ANPs for multimensional regression tasks, and the translation equivariance property of ConvCNPs for synthetic 1D regression and 2D image classification tasks. Extensive experimental results of the vNPs–SDE model show that vNPs can not only improve SDE-Net in terms of OOD detection and misclassification detection between ID and OOD datasets, but also allow it to make more efficient and accurate predictions for ID datasets with missing rates than the BBP, MC-dropout, and vanilla SDE-Net models—except for OOD detection for CIAFR10, in which BBP is superior. Hence, the ability of the BBP model in terms of OOD detection deserves further extensive experiments.

Future studies should consider the local optimal problem of deep learning models, which is also one of the sources of uncertainty that generate unstable predictions—we can find a curved path, and the parameters of DNNs on the path can produce near constant loss of deep learning. The DNNs of the proposed vNPs–SDE model are too simple, but we can apply transfer learning methods to replace the simple DNNs with the state-of-the-art ResNets, such as ResNet-18 and ResNet-34, to improve performance.

## Figures and Tables

**Table 1:** ID dataset YearPredictionMSD with MR.

Model	Missing Rate	RMSE
BBP	MR = 0.1	16.6 ± 0.1
MC-dropout		12.4 ± 0.4
ANPs–SDE-Net		9.3 ± 0.6
SDE-Net		9.3 ± 0.7
CNPs–SDE-Net		9.1 ± 0.6
BBP	MR = 0.3	20.4 ± 0.5
MC-dropout		15.2 ± 0.6
ANPs–SDE-Net		13.1 ± 0.9
SDE-Net		13.1 ± 1.0
CNPs-SDE Net		12.9 ± 0.8
BBP	MR = 0.5	23.5 ± 0.4
MC-dropout		17.5 ± 1.0
ANPs–SDE-Net		16.1 ± 1.1
SDE-Net		16.1 ± 1.2
CNPs–SDE-Net		15.8 ± 1.0
BBP	MR = 0.7	26.3 ± 1.1
MC-dropout		19.6 ± 1.2
ANPs–SDE-Net		18.5 ± 1.3
SDE-Net		18.5 ± 1.4
CNPs–SDE-Net		18.2 ± 1.2
BBP	MR = 0.9	28.8 ± 1.3
MC-dropout		21.5 ± 1.3
ANPs–SDE-Net		20.7 ± 1.4
SDE-Net		20.7 ± 1.6
CNPs–SDE-Net		20.4 ± 1.3

**Table 2:** OOD detection for regression on YearPredictionMSD + Boston Housing.

Model	#Parameters	RMSE	TNR at TPR 95%	AUROC	Detection Accuracy	AUPR In	AUPR Out
BBP	30.0K	9.5 ± 0.2	9.0 ± 1.4	56.8 ± 0.9	52.1 ± 0.7	45.3 ± 1.3	1.3 ± 0.1
MC-dropout	14.9K	8.8 ± 0.0	6.1 ± 0.5	53.0 ± 1.2	53.7 ± 0.6	99.2 ± 0.2	1.1 ± 0.1
ANPs–SDE-Net	288.5K	8.8 ± 0.0	44.4 ± 4.2	84.2 ± 1.6	75.2 ± 1.7	99.8 ± 0.0	28.8 ± 2.0
CNPs–SDE-Net	141.0K	8.9 ± 0.1	7.9 ± 1.2	59.3 ± 1.5	58.6 ± 0.9	99.2 ± 0.1	1.3 ± 0.1
SDE-Net	12.4K	8.7 ± 0.1	64.3 ± 0.6	84.1 ± 1.1	80.6 ± 0.5	99.7 ± 0.0	24.7 ± 1.0

**Table 3:** OOD detection results of the BBP, MC-dropout, ConvCNPs–SDE, and SDE-Net models on ID dataset MNIST with MR = [0.1, 0.3, 0.5, 0.7, 0.9, RMR] and OOD dataset SVHN.

MNIST with MR	Model	Classification Accuracy	TNR at TPR 95%	AUROC	Detection Accuracy	AUPR In	AUPR Out
MR = 0.1	BBP	88.76 ± 1.73	33.17 ± 5.09	87.77 ± 2.03	83.75 ± 2.32	85.85 ± 3.21	94.02 ± 3.42
	MC-dropout	98.90 ± 0.06	88.66 ± 0.04	96.22 ± 0.04	92.18 ± 0.02	89.23 ± 0.05	98.44 ± 0.03
	ConvCNPs–SDE-Net	99.32 ± 0.06	98.69 ± 0.04	99.68 ± 0.00	97.99 ± 0.02	99.01 ± 0.01	99.89 ± 0.00
	SDE-Net	98.87 ± 0.06	99.40 ± 0.03	99.86 ± 0.01	98.82 ± 0.04	99.61 ± 0.02	99.94 ± 0.01
MR = 0.3	BBP	78.37 ± 3.10	24.90 ± 3.12	82.06 ± 4.12	74.46 ± 2.27	73.86 ± 2.15	90.22 ± 2.30
	MC-dropout	93.47 ± 0.06	50.10 ± 0.06	92.01 ± 0.03	86.84 ± 0.06	84.68 ± 0.03	95.75 ± 0.05
	ConvCNPs–SDE-Net	98.94 ± 0.07	99.04 ± 0.04	99.78 ± 0.01	98.27 ± 0.06	99.34 ± 0.03	99.92 ± 0.01
	SDE-Net	94.98 ± 0.19	99.47 ± 0.02	99.70 ± 0.03	98.96 ± 0.03	99.51 ± 0.06	99.82 ± 0.04
MR = 0.5	BBP	52.50 ± 4.15	16.70 ± 2.10	70.47 ± 3.12	78.37 ± 3.10	54.43 ± 3.34	84.60 ± 2.12
	MC-dropout	75.88 ± 0.10	21.72 ± 0.09	81.64 ± 0.09	75.87 ± 0.06	70.73 ± 0.04	89.60 ± 0.05
	ConvCNPs–SDE-Net	97.45 ± 0.17	98.58 ± 0.03	99.70 ± 0.01	98.15 ± 0.05	99.17 ± 0.02	99.87 ± 0.01
	SDE-Net	80.54 ± 0.36	99.17 ± 0.05	98.45 ± 0.08	97.26 ± 0.04	98.06 ± 0.05	98.96 ± 0.07
MR = 0.7	BBP	23.71 ± 4.11	17.40 ± 2.23	66.36 ± 3.12	61.91 ± 3.23	43.01 ± 2.44	83.18 ± 2.23
	MC-dropout	46.48 ± 0.10	12.16 ± 0.16	70.32 ± 0.11	65.67 ± 0.16	53.76 ± 0.21	83.06 ± 0.15
	ConvCNPs–SDE-Net	88.96 ± 0.33	97.45 ± 0.06	99.20 ± 0.03	97.47 ± 0.07	98.03 ± 0.04	99.62 ± 0.03
	SDE-Net	49.25 ± 0.11	45.25 ± 1.36	93.53 ± 0.13	90.68 ± 0.15	92.36 ± 0.16	95.68 ± 0.12
MR = 0.9	BBP	10.08 ± 1.14	54.34 ± 2.34	84.13 ± 4.34	77.78 ± 1.44	59.63 ± 2.33	93.72 ± 1.34
	MC-dropout	17.15 ± 0.54	8.41 ± 0.44	60.38 ± 0.35	57.66 ± 0.34	38.11 ± 0.24	77.82 ± 0.36
	ConvCNPs–SDE-Net	36.54 ± 0.58	79.72 ± 0.65	96.20 ± 0.06	93.82 ± 0.14	92.86 ± 0.16	97.81 ± 0.08
	SDE-Net	14.56 ± 0.34	10.30 ± 0.49	71.82 ± 0.25	67.83 ± 0.18	64.34 ± 0.30	82.80 ± 0.23
MR = RMR	BBP	36.90 ± 10.56	26.37 ± 3.63	73.05 ± 2.26	67.85 ± 1.62	51.52 ± 2.56	87.43 ± 1.22
	MC-dropout	42.97 ± 1.60	11.26 ± 2.41	69.34 ± 1.43	64.95 ± 1.20	53.56 ± 2.62	82.43 ± 1.12
	ConvCNPs–SDE-Net	68.90 ± 11.66	95.72 ± 1.60	97.89 ± 0.85	95.91 ± 0.96	95.70 ± 1.67	98.89 ± 0.45
	SDE-Net	45.34 ± 13.66	22.73 ± 7.30	86.24 ± 5.13	83.25 ± 5.82	83.74 ± 6.71	91.00 ± 3.11

**Table 4:** Misclassification detection performance of the BBP, MC-dropout, ConvCNPs–SDE, and SDE-Net models on MNIST with MR = [0.1, 0.3, 0.5, 0.7, 0.9, RMR] and SVHN.

MNIST with MR	Model	TNR at TPR 95%	AUROC	Detection Accuracy	AUPR Succ	AUPR Err
MR = 0.1	BBP	40.78 ± 2.34	89.55 ± 0.92	82.52 ± 1.25	98.79 ± 0.22	44.14 ± 3.22
	MC-dropout	86.52 ± 1.22	95.59 ± 0.88	91.76 ± 0.68	99.93 ± 0.01	36.68 ± 1.88
	ConvCNPs–SDE-Net	92.44 ± 1.06	98.15 ± 0.12	95.01 ± 0.50	99.99 ± 0.00	31.49 ± 5.90
	SDE-Net	84.43 ± 3.22	96.75 ± 0.60	92.59 ± 0.76	99.96 ± 0.01	31.32 ± 2.89
MR = 0.3	BBP	24.90 ± 2.26	82.15 ± 2.82	75.45 ± 1.32	94.61 ± 2.34	54.26 ± 2.04
	MC-dropout	60.92 ± 1.24	91.73 ± 0.41	54.25 ± 1.51	99.21 ± 0.01	46.33 ± 1.31
	ConvCNPs–SDE-Net	85.80 ± 3.63	97.22 ± 0.42	93.00 ± 1.02	99.97 ± 0.01	35.81 ± 4.71
	SDE-Net	54.25 ± 1.51	91.11 ± 0.46	84.01 ± 0.93	99.44 ± 0.04	40.00 ± 1.75
MR = 0.5	BBP	12.17 ± 2.16	72.48 ± 1.26	68.08 ± 1.52	78.42 ± 4.25	68.32 ± 2.55
	MC-dropout	27.28 ± 0.33	82.15 ± 0.33	76.10 ± 0.23	92.85 ± 0.13	56.85 ± 0.43
	ConvCNPs–SDE-Net	72.53 ± 3.26	95.07 ± 0.51	89.36 ± 0.39	99.86 ± 0.01	36.57 ± 4.60
	SDE-Net	33.07 ± 0.53	83.79 ± 0.35	76.12 ± 0.24	95.33 ± 0.14	56.45 ± 0.83
MR = 0.7	BBP	10.17 ± 1.27	65.10 ± 1.21	65.09 ± 0.82	51.87 ± 0.57	82.44 ± 1.22
	MC-dropout	15.67 ± 0.87	73.82 ± 0.35	68.55 ± 0.32	72.23 ± 0.37	72.72 ± 0.33
	ConvCNPs–SDE-Net	43.58 ± 1.67	89.10 ± 0.16	82.10 ± 0.41	98.44 ± 0.07	48.40 ± 1.01
	SDE-Net	28.88 ± 0.97	76.12 ± 0.50	68.84 ± 0.47	75.88 ± 0.34	75.61 ± 0.76
MR = 0.9	BBP	9.21 ± 1.22	69.39 ± 1.45	64.41 ± 0.62	26.28 ± 1.46	94.27 ± 0.32
	MC-dropout	7.45 ± 0.32	59.26 ± 0.43	57.29 ± 0.12	25.06 ± 0.22	86.16 ± 0.22
	ConvCNPs–SDE-Net	15.73 ± 0.67	70.02 ± 0.70	64.73 ± 0.81	60.84 ± 1.19	77.94 ± 0.59
	SDE-Net	7.14 ± 0.42	60.91 ± 0.41	59.25 ± 0.30	22.04 ± 0.79	88.79 ± 0.25
MR = RMR	BBP	10.85 ± 2.43	70.43 ± 5.52	67.83 ± 3.42	68.10 ± 6.22	80.86 ± 3.23
	MC-dropout	34.81 ± 13.32	78.00 ± 5.40	72.60 ± 4.40	77.47 ± 9.45	76.60 ± 4.21
	ConvCNPs–SDE-Net	36.95 ± 11.08	88.19 ± 2.74	81.51 ± 2.59	94.38 ± 3.33	71.57 ± 6.18
	SDE-Net	35.11 ± 15.40	83.44 ± 6.02	76.25 ± 4.80	80.15 ± 15.44	83.92 ± 3.18

**Table 5:** Classification and OOD detection results of the BBP, MC-dropout, ConvCNPs–SDE, and SDE-Net models on ID dataset CIFAR10 with MR = [0.1, 0.3, 0.5, 0.7, 0.9, RMR] and OOD dataset SVHN.

CIFAR10 with MR	Model	Classification Accuracy	TNR at TPR 95%	AUROC	Detection Accuracy	AUPR In	AUPR Out
MR = 0.1	BBP	19.83 ± 0.45	64.31 ± 3.35	92.57 ± 2.47	86.49 ± 2.45	84.29 ± 1.55	96.46 ± 1.42
	MC-dropout	43.01 ± 0.35	3.67 ± 0.13	50.90 ± 0.15	52.50 ± 0.10	31.15 ± 0.16	71.50 ± 0.05
	ConvCNPs–SDE-Net	78.66 ± 0.20	4.46 ± 0.31	59.97 ± 0.16	59.33 ± 0.11	42.23 ± 0.15	75.54 ± 0.07
	SDE-Net	23.23 ± 0.25	1.57 ± 0.10	37.79 ± 0.13	50.36 ± 0.06	24.04 ± 0.14	63.41 ± 0.06
MR = 0.3	BBP	19.64 ± 0.15	59.53 ± 3.43	92.62 ± 1.89	86.50 ± 1.55	86.45 ± 2.45	96.36 ± 1.45
	MC-dropout	24.91 ± 0.25	1.87 ± 0.15	40.81 ± 0.35	50.01 ± 0.26	23.62 ± 0.12	65.83 ± 0.25
	ConvCNPs–SDE-Net	76.46 ± 0.20	4.25 ± 0.37	58.31 ± 0.30	57.89 ± 0.20	40.55 ± 0.18	74.71 ± 0.22
	SDE-Net	13.11 ± 2.89	4.61 ± 0.25	52.28 ± 0.34	52.55 ± 0.15	31.46 ± 0.12	72.74 ± 0.30
MR = 0.5	BBP	19.44 ± 0.25	46.25 ± 4.26	87.53 ± 3.72	81.05 ± 3.72	77.70 ± 3.38	93.95 ± 1.68
	MC-dropout	18.49 ± 0.19	2.22 ± 0.15	40.79 ± 0.19	50.00 ± 0.15	23.42 ± 0.17	66.10 ± 0.16
	ConvCNPs–SDE-Net	72.21 ± 0.26	3.57 ± 0.19	55.08 ± 0.28	55.35 ± 0.13	37.36 ± 0.15	72.98 ± 0.22
	SDE-Net	10.33 ± 0.06	4.97 ± 0.08	50.74 ± 0.26	50.95 ± 0.17	29.18 ± 0.21	72.33 ± 0.13
MR = 0.7	BBP	18.39 ± 0.52	21.77 ± 4.09	77.28 ± 3.11	70.66 ± 2.47	67.15 ± 2.63	87.66 ± 2.29
	MC-dropout	14.61 ± 0.18	2.76 ± 0.28	42.66 ± 0.22	50.00 ± 0.13	24.26 ± 0.23	67.36 ± 0.14
	ConvCNPs–SDE-Net	62.47 ± 0.49	2.50 ± 0.06	48.70 ± 0.28	52.08 ± 0.05	31.64 ± 0.14	69.60 ± 0.13
	SDE-Net	10.10 ± 0.08	4.91 ± 0.28	49.86 ± 0.50	50.27 ± 0.19	27.85 ± 0.31	72.05 ± 0.34
MR = 0.9	BBP	14.60 ± 0.67	1.10 ± 0.40	55.31 ± 2.84	65.55 ± 1.23	54.23 ± 2.43	68.97 ± 0.93
	MC-dropout	12.01 ± 0.16	2.81 ± 0.11	43.53 ± 0.06	50.00 ± 0.07	24.45 ± 0.04	67.91 ± 0.16
	ConvCNPs–SDE-Net	36.48 ± 0.43	1.29 ± 0.01	37.86 ± 0.39	50.49 ± 0.05	23.48 ± 0.30	64.32 ± 0.18
	SDE-Net	10.18 ± 0.06	4.91 ± 0.18	49.40 ± 0.06	50.19 ± 0.12	27.02 ± 0.05	71.92 ± 0.11
MR = RMR	BBP	16.37 ± 0.38	15.04 ± 5.27	70.19 ± 1.22	65.72 ± 0.64	59.93 ± 0.21	83.36 ± 1.87
	MC-dropout	14.78 ± 0.08	2.31 ± 0.25	41.87 ± 0.35	50.00 ± 0.15	23.72 ± 0.23	66.80 ± 0.33
	ConvCNPs–SDE-Net	56.04 ± 4.08	1.98 ± 0.24	46.79 ± 2.04	51.80 ± 0.48	30.41 ± 1.90	68.45 ± 0.98
	SDE-Net	10.18 ± 0.05	4.82 ± 0.26	50.01 ± 0.36	50.42 ± 0.22	28.00 ± 0.28	72.03 ± 0.27

**Table 6:** Misclassification detection performance of the BBP, MC-dropout, ConvCNPs-SDE, and SDE-Net models on CIFAR10 with MR = [0.1, 0.3, 0.5, 0.7, 0.9, RMR] and SVHN.

CIFAR10 with MR	Model	TNR at TPR 95%	AUROC	Detection Accuracy	AUPR Succ	AUPR Err
MR = 0.1	BBP	10.97 ± 0.66	58.75 ± 0.62	56.19 ± 0.70	25.69 ± 0.32	85.16 ± 0.77
	MC-dropout	10.89 ± 0.60	67.19 ± 0.12	63.17 ± 0.23	61.69 ± 0.13	69.60 ± 0.24
	ConvCNPs–SDE-Net	27.47 ± 0.80	83.77 ± 0.18	77.33 ± 0.26	94.88 ± 0.12	54.12 ± 0.64
	SDE-Net	21.85 ± 1.60	72.51 ± 0.29	66.64 ± 0.35	44.85 ± 0.58	88.73 ± 0.21
MR = 0.3	BBP	9.84 ± 0.34	56.17 ± 0.24	54.57 ± 0.34	24.77 ± 0.14	84.32 ± 0.67
	MC-dropout	6.47 ± 0.34	58.22 ± 0.26	56.90 ± 0.46	32.83 ± 0.66	78.92 ± 0.24
	ConvCNPs–SDE-Net	26.96 ± 0.83	82.70 ± 0.24	76.04 ± 0.37	93.83 ± 0.17	55.70 ± 0.50
	SDE-Net	8.61 ± 1.24	65.01 ± 0.68	63.04 ± 0.57	22.85 ± 0.88	91.82 ± 0.34
MR = 0.5	BBP	8.19 ± 0.02	55.73 ± 0.68	55.17 ± 0.70	23.32 ± 0.43	83.74 ± 0.02
	MC-dropout	6.11 ± 0.14	57.18 ± 0.23	56.69 ± 0.22	24.32 ± 0.26	84.06 ± 0.34
	ConvCNPs–SDE-Net	23.55 ± 0.79	80.31 ± 0.16	73.78 ± 0.33	91.32 ± 0.05	57.15 ± 0.57
	SDE-Net	5.89 ± 1.22	53.64 ± 0.83	53.40 ± 0.19	13.31 ± 0.32	90.45 ± 0.33
MR = 0.7	BBP	9.00 ± 0.09	53.62 ± 0.66	53.73 ± 0.61	20.29 ± 1.04	84.15 ± 0.25
	MC-dropout	5.89 ± 0.78	56.51 ± 0.58	56.08 ± 0.38	18.90 ± 0.22	87.23 ± 0.38
	ConvCNPs–SDE-Net	18.24 ± 0.69	75.85 ± 0.39	69.81 ± 0.40	84.37 ± 0.38	61.18 ± 1.22
	SDE-Net	5.05 ± 0.98	50.28 ± 0.51	51.17 ± 0.40	10.67 ± 0.44	89.87 ± 0.28
MR = 0.9	BBP	5.06 ± 0.13	53.45 ± 2.20	53.78 ± 1.96	16.89 ± 1.98	86.25 ± 0.14
	MC-dropout	5.73 ± 0.78	55.93 ± 0.35	55.23 ± 0.28	14.60 ± 0.75	89.49 ± 0.23
	ConvCNPs–SDE-Net	11.53 ± 0.48	66.46 ± 0.45	62.43 ± 0.48	56.21 ± 0.53	74.77 ± 0.72
	SDE-Net	4.81 ± 0.88	50.93 ± 0.40	51.68 ± 0.36	11.26 ± 0.68	89.86 ± 0.25
MR = RMR	BBP	8.12 ± 0.17	55.30 ± 1.18	53.92 ± 0.85	19.66 ± 0.15	86.11 ± 0.74
	MC-dropout	6.66 ± 0.58	56.51 ± 0.88	55.59 ± 0.83	19.04 ± 0.55	87.32 ± 0.25
	ConvCNPs–SDE-Net	16.33 ± 1.57	75.42 ± 1.58	69.77 ± 1.39	80.73 ± 3.76	66.10 ± 2.00
	SDE-Net	5.34 ± 0.77	51.45 ± 0.94	51.74 ± 0.89	11.69 ± 0.56	90.21 ± 0.24

## Data Availability

Datasets are open access and available on References [33,41,42].

## Abbreviations

The following abbreviations are used in this manuscript:

SDE-Net	Neural stochastic differential equation model
DNNs	Deep neural networks
ID	In-distribution
OOD	Out-of-distribution
NPs	Neural processes
vNPs	Vanilla neural processes or neural process variants
ConvCNP	Convolutional conditional neural process
CNPs	Conditional neural processes
ANPs	Attentive neural processes
BNNs	Bayesian neural processes
PCA	Principal component analysis
GPs	Gaussian processes
MLP	Multilayer perceptron
CNNs	Convolutional neural networks
ODE-Net	Neural ordinary differential equation
Conv2d	Two-dimensional convolution
RKHS	Reproducing kernel Hilbert space
ResNet	Residual networks
ELBO	Evidence lower bound
KL	Kullback–Leibler divergence
